# Superior vena cava syndrome induced by lung hyperinflation in chronic obstructive pulmonary disease: a case report

**DOI:** 10.1186/s13256-023-04256-7

**Published:** 2023-12-01

**Authors:** Nobuhiro Kanaji, Naoki Watanabe, Takuya Inoue, Hitoshi Mizoguchi, Kosuke Sakamoto, Yuta Komori, Kosuke Kawada, Norimitsu Kadowaki

**Affiliations:** 1 Division of Hematology, Rheumatology and Respiratory Medicine, Department of Internal Medicine, Faculty of Medicine, Kagawa University, 1750-1 Ikenobe, Miki-Cho, Kita-Gun, Kagawa 761-0793 Japan; 2 Department of Cardiovascular Surgery, Faculty of Medicine, Kagawa University, Kita-Gun, Kagawa Japan

**Keywords:** Emphysema, Chronic obstructive pulmonary disease, Superior vena cava syndrome, Case report

## Abstract

**Background:**

Superior vena cava syndrome is rarely attributed to chronic obstructive pulmonary disease.

**Case presentation:**

We present the case of an 82-year-old Japanese man who experienced gradually progressive dyspnea on exertion. His physical examination revealed small vascular dilatations on his chest and upper abdominal skin characterized by blood flow from head to leg, indicating superior vena cava syndrome. Radiographic findings included lung hyperinflation with a drop-like heart on chest X-ray, and emphysematous changes on computed tomography. The superior vena cava appeared extremely narrow and slit-like, with no adjacent mass or giant bulla. Pulmonary function testing indicated a forced expiratory volume in 1 second of 0.82L (44.4% of predicted value) and a forced expiratory volume in 1 second/forced vital capacity of 31.29%. A diagnosis of chronic obstructive pulmonary disease was made. We discuss how longitudinal forces can narrow the superior vena cava, particularly when it protrudes toward the lung field due to its anatomical location in the upper mediastinum. The absence of mediastinal adipose tissue may render the superior vena cava susceptible to compression, resulting in a loss of its typical columnar structure. The protrusion of the superior vena cava toward the lung field may be a contributing factor to superior vena cava narrowing in chronic obstructive pulmonary disease.

**Conclusion:**

This case represents the first reported instance of superior vena cava syndrome associated with chronic obstructive pulmonary disease, characterized by lung hyperinflation, in the absence of a giant bulla.

## Background

Superior vena cava (SVC) syndrome is a collection of clinical signs and symptoms that manifest when the SVC is either partially or completely obstructed [1]. It can be readily identified through clinical evaluation [2]. SVC syndrome can be attributed to various diseases, including thoracic malignancies. Consequently, additional investigations such as computed tomography (CT) are necessary to determine the underlying cause of SVC syndrome. Notably, chronic obstructive pulmonary disease (COPD) as a causative factor for SVC syndrome is rare. Only two cases of SVC syndrome resulting from giant bullous lesions have been reported [3, 4]. In this case report, we describe a patient with COPD who presented with SVC syndrome attributed to lung hyperinflation in the absence of a giant bulla.

## Case presentation

**Fig. 1:**
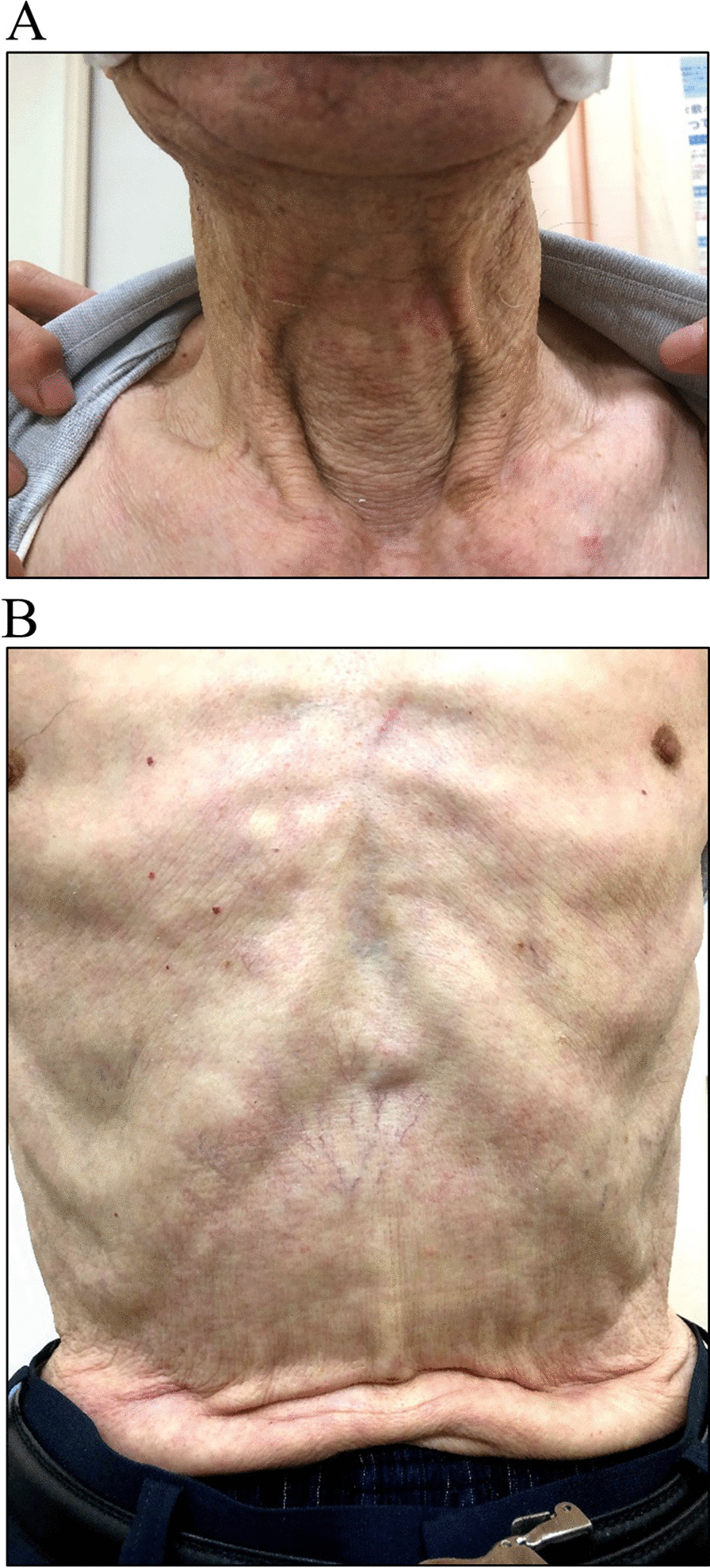
Pictures of (**A**) neck and (**B**) trunk of the body in the patient

**Fig. 2:**
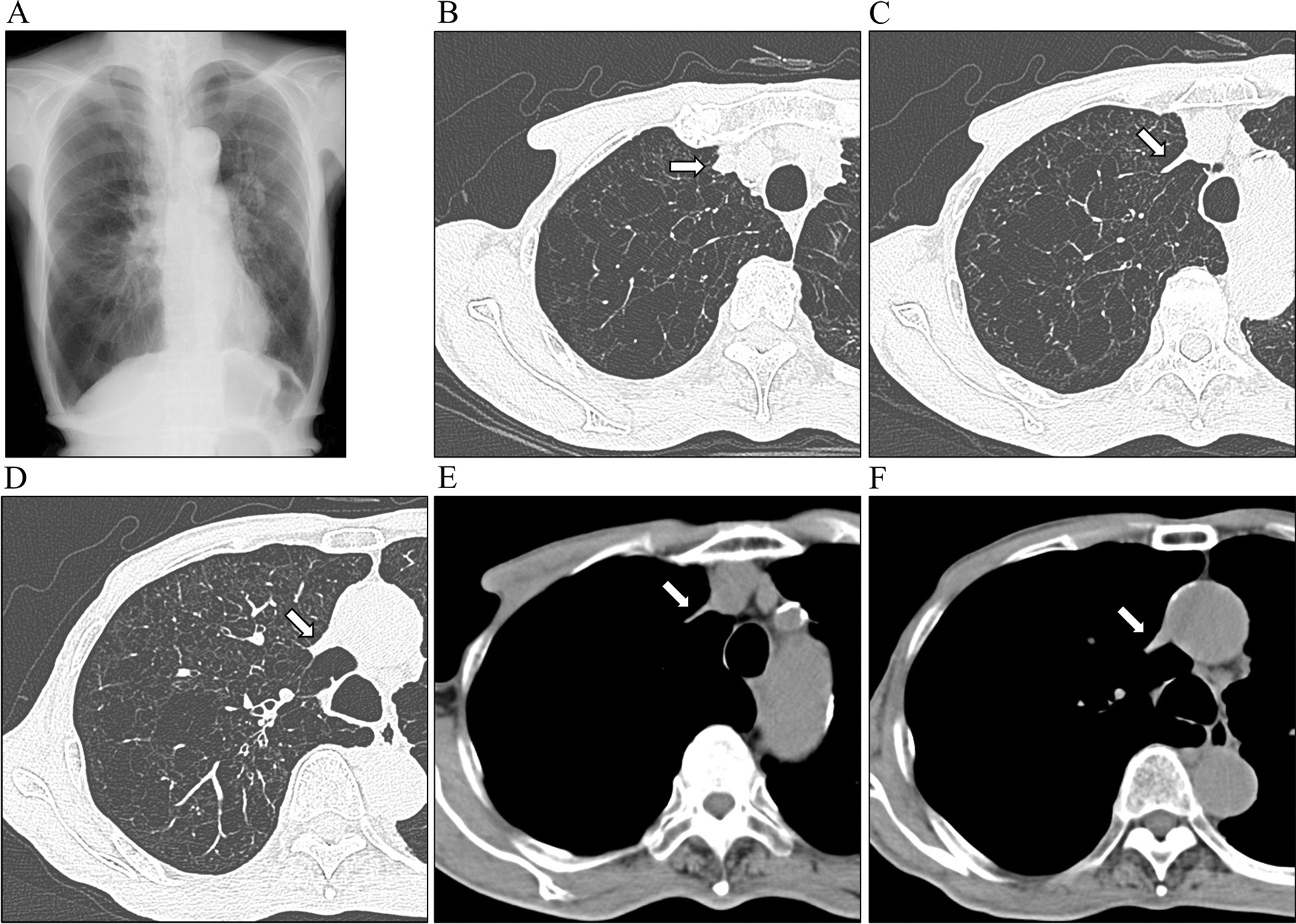
**A** Chest X-ray showing hyperinflation of the lung field. **B–F** Chest computed tomography showing emphysematous change. Arrows indicate the superior vena cava, which is partly extremely narrow

An 82-year-old Japanese man presented to the hospital with a history of progressive dyspnea on exertion (mMRC grade 2) that had been ongoing for several years. He had worked as a fisherman and had been a one-pack-a-day cigarette smoker for over 50 years. He never consumed alcohol and had no previous history of malignancies or cardiovascular diseases. He has no history of catheterization such as central venous catheters or irradiation. Upon examination, his height measured 164.7 cm and he weighed 48.5 kg, resulting in a body mass index of 17.9. He was found to have pursed-lips breathing with hypertrophic sternocleidomastoid muscles. However, there were no noticeable signs of swelling in his neck, face, or upper extremities (Fig. 1A). Additionally, we observed intercostal enlargement and reduced thoracic movement during respiration. Small vascular dilatations were visible on his chest and upper abdomen (Fig. 1B), with blood flow noticed in the direction of head to leg, a characteristic sign of SVC syndrome. Chest radiography revealed hyperinflation with a drop-like appearance of the heart (Fig. 2A), while CT scans depicted extensive emphysematous changes (Fig. 2B–F). Notably, the SVC exhibited partial narrowing in a slit-like configuration (Fig. 2C, indicated by arrow). No mass lesions compressing the SVC were identified in the lung fields or mediastinum, and there were no giant bullae adjacent to the SVC. Pulmonary function testing yielded the following results: vital capacity (VC) of 2.88 (88.3% of the predicted value), forced expiratory volume in one second (FEV1) of 0.82L (44.4% of the predicted value), FEV1% (FEV1/forced VC) of 31.29%, and fractional exhaled nitric oxide level of 14 ppb. The blood D-dimer level was within normal range, indicating an absence of thrombotic occlusion. Hemoglobin in the blood was 11.8 g/dL. Arterial blood gas analysis demonstrated a pH of 7.419, PaCO_2_ of 40.8 mmHg, and PaO_2_ of 57.8 mmHg. As a result, the patient received a diagnosis of COPD. He was started on a long-acting muscarinic antagonist and a long-acting beta-2 agonist. After 3 months, there was a slight improvement in his dyspnea. However, physical examination findings related to SVC syndrome remained the same, and the narrowing of the SVC persisted on CT images (not shown).

## Discussion and conclusion

To the best of our knowledge, this is the first reported case of SVC syndrome attributed to lung hyperinflation accompanied by COPD.

According to a previous report, SVC syndrome has been predominantly associated with malignancies, accounting for 70% of cases, while benign conditions make up the remaining 30% [5]. Among malignancies, non-small cell lung cancer represents the most prevalent etiology, accounting for nearly 50% of cases. Other malignant causes include small cell lung cancer, malignant lymphomas, germ cell tumors, malignant mesotheliomas, and thymic tumors [5, 6]. Benign causes of SVC syndrome encompass device-related factors (central venous catheters, pacemakers, defibrillators, and indwelling hemodialysis catheters), contributing to approximately 25% to 30% of total SVC cases. Other benign causes include radiation fibrosis, infection, retrosternal thyroid enlargement, aortic aneurysms, benign tumors, mediastinal hematoma, sarcoidosis, etc. Prior to this case, there have been no documented instances of SVC syndrome directly associated with COPD or emphysema, except for two cases involving a giant bullous lesion [3, 4], where the syndrome was linked to the compression of the SVC by these bullae.

The most common signs and symptoms associated with SVC syndrome include neck, upper extremity, and facial swelling, dyspnea, chest pain, cough, weight loss, jugular venous distension, and dilated collateral chest veins [2]. These symptoms typically manifest gradually. It is worth noting that the rate of symptom onset correlates with symptom severity, as a slower progression of SVC obstruction allows time for the development of collateral circulation [2]. In the case presented here, the patient experienced a gradual progression of dyspnea and weight loss. Notably, there was no observable swelling in the neck and upper extremities, but dilated collateral chest veins were evident. These findings suggest a gradual progression of lung hyperinflation. While there are potential treatment options available for SVC syndrome, such as SVC stenting, endobronchial valve placement, or other surgical interventions, these procedures were not pursued in this case. Several factors influenced this decision, including the chronic nature of the disease, the absence of acute symptoms, partial SVC stenosis rather than complete obstruction, and the presence of collateral vessels.

**Fig. 3:**
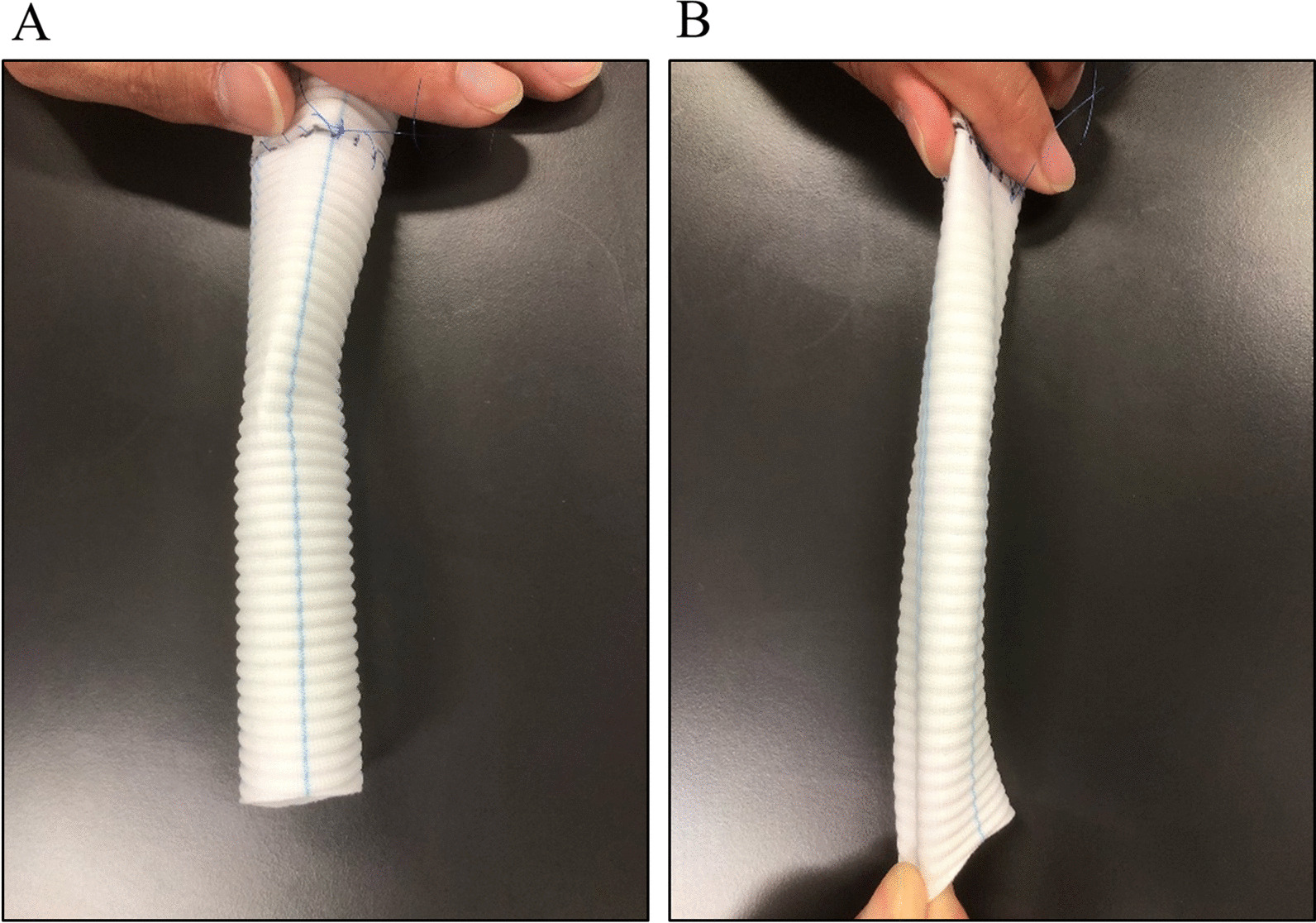
The artificial blood vessel. **A** The natural state. **B** The state pulled in lengthwise direction

The radiographic signs of a drop-like appearance of the heart and the flattened, low configuration of the diaphragm are well-documented indicators of lung hyperinflation [7]. To illustrate this concept, consider the behavior of a soft tube when it is elongated longitudinally; its lumen narrows. Artificial blood vessels are no exception to this phenomenon (Fig. 3). However, an artificial blood vessel with a jagged structure would likely deform more easily compared with the actual SVC. In materials with high elasticity, such as the stretchy material mentioned, when a tube is elongated, the force is typically concentrated primarily on the localized area where the stretching occurs, rather than being evenly distributed throughout the entire tube. The actual SVC would distribute any pulling force more uniformly across its structure compared with artificial blood vessels.

Conversely, it is important to note that not all cases of lung hyperinflation lead to SVC syndrome. In this context, the anatomical location of the SVC is considered a significant factor contributing to the development of the slit-like configuration observed in SVC syndrome. The SVC typically resides in the upper mediastinum, primarily on the right side, and in some instances, it may even protrude towards the lung field, as seen in the current case. When positioned in this manner, the SVC appears to be more susceptible to compression because it lacks the protective cushioning of mediastinal adipose tissue, making it prone to deformation and loss of its usual columnar blood vessel structure.

This case illustrates that hyperinflation of the lung, resulting from emphysematous change, can induce SVC syndrome, even in the absence of giant bullae. The positioning of the SVC extending towards the lung field may indeed be a contributing factor to SVC narrowing in individuals with COPD.

## Data Availability

There is no additional available data other than Figures.

## Abbreviations

COPD: Chronic obstructive pulmonary disease
CT: Computed tomography
FEV1: Forced expiratory volume in 1 second
SVC: Superior vena cava
VC: Vital capacity
